# More frequent, more costly? Health economic modelling aspects of monitoring glaucoma patients in England

**DOI:** 10.1186/s12913-016-1849-9

**Published:** 2016-10-22

**Authors:** Trishal Boodhna, David P. Crabb

**Affiliations:** Division of Optometry and Visual Science, School of Health Sciences, City, University of London, Northampton Square, London,, EC1V 0HB UK

**Keywords:** Glaucoma, Health economic model, QALY, Visual fields, Health service delivery, Visual impairment

## Abstract

**Background:**

Chronic open angle glaucoma (COAG) is an age-related eye disease causing irreversible loss of visual field (VF). Health service delivery for COAG is challenging given the large number of diagnosed patients requiring lifelong periodic monitoring by hospital eye services. Yet frequent examination better determines disease worsening and speed of VF loss under treatment. We examine the cost-effectiveness of increasing frequency of VF examinations during follow-up using a health economic model.

**Methods:**

Two different VF monitoring schemes defined as *current practice* (annual VF testing) and *proposed practice* (three VF tests per year in the first 2 years after diagnosis) were examined. A purpose written health economic Markov model is used to test the hypothesis that cost effectiveness improves by implementing *proposed practice* on groups of patients stratified by age and severity of COAG. Further, a new component of the model, estimating costs of visual impairment, was added. Results were derived from a simulated cohort of 10000 patients with quality-adjusted life years (QALYs) and incremental cost-effectiveness ratios (ICERs) used as main outcome measures.

**Results:**

An ICER of £21,392 per QALY was derived for *proposed practice* improving to a value of £11,382 once savings for prevented visual impairment was added to the model. *Proposed practice* was more cost-effective in younger patients. *Proposed practice* for patients with advanced disease at diagnosis generated ICERs > £60,000 per QALY; these cases would likely be on the most intensive treatment pathway making clinical information on speed of VF loss redundant. Sensitivity analysis indicated results to be robust in relation to hypothetical willingness to pay threshold identified by national guidelines, although greatest uncertainty was allied to estimates of implementation and visual impairment costs.

**Conclusion:**

Increasing VF monitoring at the earliest stages of follow-up for COAG appears to be cost-effective depending on reasonable assumptions about implementation costs. Our health economic model highlights benefits of stratifying patients to more or less monitoring based on age and stage of disease at diagnosis; a prospective study is needed to prove these findings. Further, this works highlights gaps in knowledge about long term costs of visual impairment.

## Background

Chronic open-angle glaucoma (COAG) is an age-related eye disease of the optic nerve [1]. Early onset COAG is typically asymptomatic but as it advances so does the risk of irreversible loss of sight. Visual impairment in COAG, manifesting as loss of visual field (VF), may be associated with restricted mobility, falls, motor vehicle accidents and reduced quality of life [2, 3]. Furthermore, COAG is the second major cause for blind registration in the United Kingdom (UK) [4]. In the UK around 2 % of people over 40 years have COAG, rising to almost 10 % in people over 75 years [5]. With an aging population the number of people affected by COAG will increase. Fortunately, most patients respond to treatment and only a minority go on to develop visual impairment [6]. Yet, once diagnosed, all patients require lifelong clinical follow-up so that any worsening of disease can be detected and treatment intensified accordingly. Monitoring COAG in secondary care (hospital eye services) focuses upon the evaluation of the VF, assessment of the optic nerve and measurement of the elevation of intraocular pressure (IOP). The latter is the only treatable risk factor for VF deterioration (progression) [7]. In some patients only modest pharmacological treatment may be required since progression is static or slow, while in others it may be very difficult to control rapidly progressive disease.

The lifelong nature of glaucoma follow-up means there are long-term economic implications. Glaucoma patients cause considerable direct costs to the UK National Health Service (NHS) due to monitoring expenditures, costs of medication, procedures and outpatient clinic visits. In England and Wales alone, it has been estimated that there are more than one million glaucoma related visits to hospital eye service every year [5]. In addition to this direct burden, considerable indirect costs are also incurred as a consequence of progression to potential visual impairment and blindness [8, 9]. Significant trends between the costs and severity of disease have been reported [10–12]. As such, a potential economic argument accompanies the clinical reasoning for increased monitoring of patients with glaucoma in order to potentially reduce the number of patients progressing to serious sight loss [13].

In short, VF testing aims to locate damaged areas in a patient’s field of vision using a technique, called perimetry, that systematically measures the patient’s ability to identify the presence of a small spot of light [14]. The computerised instrument produces a map of VF loss in each eye. Changes in these maps between follow-up visits can be used to assess VF progression or stability. Patients with fast VF progression are in greater danger of visual impairment, in a given time-frame, than patients with slow progression. Therefore, VF monitoring is an important component in the management of a patient. However, VF testing produces variable measurements which necessitate frequent monitoring or a considerable period of time to precisely detect true disease progression. Several studies have shown increasing the frequency of VF testing (more examinations per year) at different stages of follow-up leads to earlier detection of progression [13, 15–18]. Simply put, an adequate number of VF tests must be performed over a given period in order to separate true disease progression from the measurement variability inherent in VF data. Nevertheless, frequency of monitoring presents a dilemma for health service delivery for patients with COAG: if VF changes are not detected early enough, because of infrequent testing, there might be long term costs associated with the disease progression following inadequate treatment; on the other hand, if patients are examined too often there is increased pressure on clinic resources. It is this dilemma that is examined in this report.

The best way to examine different monitoring schemes would be with a randomised clinical trial. No such study has been performed and it would likely be substantial and costly. The first step ought to involve some modelling of existing data. We previously examined the cost-effectiveness of using different monitoring intervals to detect VF progression rates in all newly-diagnosed COAG patients using a health economic model developed for the purpose [19]. Two different VF monitoring schemes defined as *current practice* (annual VF testing) and *proposed practice* (three VF tests per year in the first 2 years after diagnosis) were examined. We now update aspects of the model to examine the hypothesis that cost effectiveness improves by implementing *proposed practice* on groups of patients stratified by age and severity of glaucoma at diagnosis. Further, a new component of the model, estimating costs of visual impairment, is added. We hypothesise that *proposed practice* applied to some groups of patients will yield improved clinical information and therefore increase the cost-effectiveness of clinical care. The outcome of this economic evaluation could potentially provide information to assist decision-makers in the allocation of the available resources so that benefits can be maximised; it could also be used to help design an appropriate prospective study on frequency of monitoring in glaucoma.

## Methods

In this section we first outline the national guidelines for determining cost-effectiveness of clinical intervention. Then we outline the difference between *current practice* and *proposed practice* for VF follow-up in COAG. Next we briefly describe our health economic model, since the details are published elsewhere [19]; this review includes a brief description of how treatment pathways are adapted given what we define as *perfect information* about observed disease progression that might be better afforded by the increased monitoring in *proposed practice*. New updates to the published model, including costs for visual impairment, are also described. The model is then used in a novel fashion to experiment with applying *proposed practice* to groups of patients stratified by age and disease severity at diagnosis. Finally, in sensitivity analysis, we explore the impact of changing model parameters.

### National guidelines for cost-effectiveness of clinical intervention

In England and Wales, the National Institute for Health and Care Excellence (NICE) is responsible for establishing evidence based guidelines for clinical practice and recommendations about resource allocation within the NHS. NICE also attempts to assess the cost-effectiveness of potential expenditures within the NHS. For example, benefits associated with different interventions are typically assessed using the quality adjusted life year (QALY) and the derivation of incremental cost-effectiveness ratios (ICERs) [20]; these identify the cost with which an extra QALY is produced by the new intervention which can then be compared against the willingness to pay for these units of health benefit in the NHS. ICERs of £20,000 or lower per QALY are thought to be acceptable, with ICERs between £20,000 and £30,000 also having a high probability of acceptance by NICE [21, 22].

### Definition of *current practice* and *proposed practice*

Recommendations for frequency of follow-up for patients diagnosed with COAG in England and Wales (NHS) have been set by NICE [5]. Following diagnosis, long term monitoring of IOP, assessment of the optic nerve and the VF is required. Monitoring intervals are recommended according to risk of progression, control of IOP and treatment. These intervals range from 3 to 12 months and more detail can be found elsewhere [5]. However, VF monitoring intervals assigned by clinicians for hypothetical patient scenarios have been shown to be variable [23]. In addition, audit data from six hospitals in England showed most patients only get two or three VF examinations in the first two years after diagnosis [24]. More recently we examined a very large number of COAG patient appointments in electronic patient records from four different centres in England and found most patients get an annual VF examination only [25]. For these reasons we make the simplifying assumption that after diagnosis of COAG annual testing of the VF is *current practice*.

**Fig. 1:**
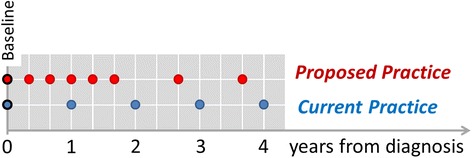
Schematic illustrating the time points at which VF examinations could be performed under *current practice* and *proposed practice* up to 4 years. Proposed practice detects progression earlier but comes at the costs of more testing

Several studies have suggested increasing the frequency of VF examinations, at different points in follow-up, may lead to better detection of glaucoma progression [13, 15–18]. Specifically it has been recommended that newly-diagnosed patients should undergo VF testing three times per year in the first 2 years after diagnosis [13]. This frequency of testing identifies rapidly progressing eyes with greater certainty than if annual testing was implemented and can help characterise clinically important information about speed (rate) of disease progression. The latter, coupled with a patient’s age, is important in order to determine lifetime risk of visual impairment and ought to lead to better clinical management decisions. The recommendation of six VF examinations in the first 2 years after diagnosis, recently adopted in the European Glaucoma Society (EGS) guidelines on patient examination for COAG [26], is defined as *proposed practice* (see Fig. 1).

(It is important to note current EGS guidelines simultaneously recognize there is no solid evidence for optimum monitoring schemes for patients with COAG. Furthermore some evidence considered by the guidelines also questions the value of more frequent monitoring [26]).

For the purpose of this work, VF progression was defined as a reduction in the mean deviation (MD) index (dB/year). MD is conventionally used in the clinic and in clinical trials; it is a summary measure of the overall reduction in VF sensitivity relative to a group of healthy age-matched observers with more negative values indicating more vision loss [14]. Time period (years) required to detect various rates of MD change in VFs were calculated via extensive simulations and the results of these are published elsewhere [19, 27]. In short, detection time of disease progression is potentially delayed, on average, by about two years with *current practice* compared to *proposed practice*. This difference in detection time might provide more timely intervention but at the cost of more testing, and hospital visits, in the initial two years of follow-up.

### Health economic model

The health economic model was purpose written in Microsoft Excel, and is described extensively elsewhere in an open-access National Institute for Health Research (NIHR) report [19]. From this point we refer to this as the frequency of VF monitoring (FVFM) model. Now we summarise the main features of the FVFM model and only detail aspects that we have newly updated.

FVFM uses a Markov model to compare *proposed practice* against *current practice* for patients with newly diagnosed COAG during a 25-year horizon. Markov models are commonly used for quantifying the costs and health consequences of patients moving through different disease stages over time [20, 28, 29].

**Fig. 2:**
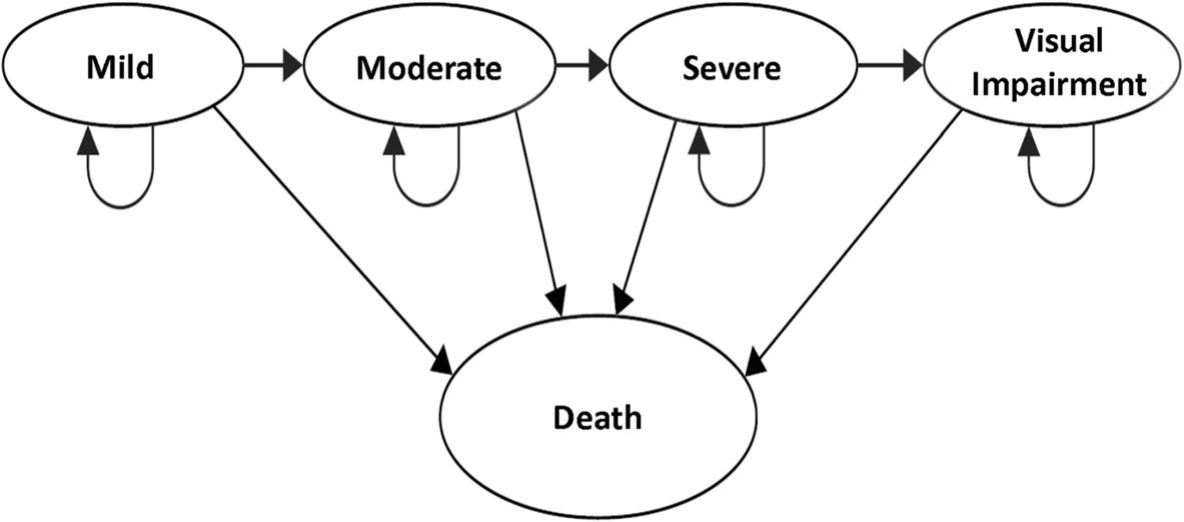
A schematic of the Markov Model for a glaucoma disease ’pathway’

In the model, patients can start in any one of four states of severity of disease at diagnosis. We assume that one cycle through the Markov model is 1-year long. In each cycle through the model, the costs and utilities are calculated for each cohort of patients. In a particular model cycle, patients can remain within their existing health state, or progress towards a worse health state. Progression towards a worse disease severity is the only possible transition because vision loss in COAG is irreversible. It is also assumed that patients move sequentially and cannot skip states due to the slow evolution of the disease. Patients may also leave the model and move into an absorbing state (‘Death’). All-cause mortality is incorporated throughout every cycle of the Markov model. Data was sourced on life expectancy and annual membership of the model was adjusted to account for a certain proportion of patients leaving the model due to all-cause mortality (See Fig. 2).

The disease process in glaucoma is a complex multivariable one and long-term outcomes for individuals are often unpredictable. For our model, we only conceptualise disease progression and its treatment as they manifest in clinical practice. First, the model is only applicable to patients that have a diagnosis of COAG as defined by NICE and is not relevant to patients with a diagnosis of ocular-hypertension or others that are at risk of glaucoma. Next, VF damage alone is used as a proxy for glaucoma disease severity. Disease progression is modelled by means of the speed (rate) at which the MD worsens. Then, we make the simplifying assumption that the effect of treatment lowers IOP, which in turn affects the VF progression rate and reduces the movement between the disease states. The model then assesses the impact of being able to institute treatment decisions earlier because of the better clinical information afforded by the *proposed practice* compared to *current practice*.

In order to reduce model complexity and allow simple decisions about treatment pathways we assume that a patient can be characterised according to four categorical variables at the point of diagnosis of COAG:

- *Age* (younger patient; older patient)
- *Severity of disease* (mild; moderate; severe; visually impaired)
- *Rate of progression* (stable; slow; medium; fast)
- *Risk of progression* (high risk; typical risk)

*Age* of patient is reduced to a dichotomous variable – the modelled younger and older patient has an age of 50 and 70 years at diagnosis respectively, making up 28.2 and 71.8 % of the cohort respectively. The rationale for these values and distribution is detailed in the description of the FVFM model. *Severity of Disease* (health states) was defined according to a commonly used classification of MD [30]. Conveniently this scheme has been used in previous health economic models of glaucoma health service delivery and, importantly for our purposes, allows for use of utilities reported elsewhere [31–33]. *Mild disease* is defined as VF loss with an MD better than -6 dB. *Moderate disease* is defined as VF loss with an MD between -6 dB and -12 dB. *Severe disease* is defined as VF loss with an MD between -12 dB and -20 dB; very few of these patients would satisfy the visual field component for legal fitness to drive for example [34]. Of course, people function visually with both eyes and the better seeing eye is the best estimate of visual function [35]. Therefore, these levels of disease severity were required to exist in the patient’s better eye (defined as the eye with the better MD) since this best reflects the patients visual morbidity [36]. Patients with MDs worse than -20 dB were classified as *visually impaired* [12, 37].

*Rate (Speed) of progression* in an individual patient can be estimated from MD loss per year for patients using linear regression of MD against time [25]. The more negative the rate the faster the progression speed. These rates are categorised as stable (≥0 dB/year), slow (between 0 and -0.5 dB/year), medium (between -0.5 and -1.5 dB/year) or fast (worse than -1.5 dB/year). It is important to note that observed rate of progression is only available to the clinician in our model when sufficient VFs have been done to precisely detect it - this is termed ‘*perfect information’*. It is therefore this variable that varies between proposed and current practice.

*Risk of progression* in COAG is nebulous and multifactorial. Apart from level of IOP, risk of progression is composed among other factors of baseline diagnosis of exfoliation syndrome, decreased corneal thickness, structural changes to the optic nerve head and the retinal nerve fibre layer and co-morbidity of other eye diseases [1]. For our model we took the simplifying step of denoting patients to have *high* progression risk or *typical* progression risk and the input parameters were taken from the FVFM model.

Consequently, at diagnosis of COAG, there are 64 types of ‘patients’ based on the permutations of the initial model parameters. The relative proportions belonging to each group were estimated from data observed in glaucoma clinics in England. For this report figures for severity of disease and rate of progression were newly updated following recently published work and these are summarised in Table 1 [25, 38].

**Table 1:** Parameters for our updated model were estimated from a retrospective analysis of an electronic patient record containing 473,252 VFs downloaded in 2012 from Moorfields Eye Hospital in London; Cheltenham General Hospital Gloucestershire Eye Unit; Queen Alexandra Hospital in Portsmouth and the Calderdale and Huddersfield NHS Foundation Trust

Parameter	Stratification	50 y/o	70 y/o
Progression Rate Distribution^a^	Stable (0 dB/year)	46.7 %	37.9 %
	Slow (-0.25 dB/year)	37.8 %	36.6 %
	Medium (-1 dB/year)	12.5 %	19.1 %
	Fast (-1.5 dB/year)	3.0 %	6.4 %
Health State Distributions^b^	Mild (> -6 dB)	83.0 %	79.8 %
	Moderate (-6 dB to -12 dB)	10.8 %	15.0 %
	Severe (-12 dB to -20 dB)	5.6 %	4.1 %
	Visually Impaired (<-20 dB)	0.6 %	1.1 %
Initial Damage^c^	Mild	−3.1 dB	−3.1 dB
	Moderate	−8.3 dB	−8.4 dB
	Severe	−15.5 dB	−15.4 dB
	Visually Impaired	−24.0 dB	−23.6 dB

Baseline progression rate and existing damage in the better eye were revised following methods used in two studies using this dataset to examine levels of rates of loss and existing disease severity distributions at diagnosis (^a^ = [25] ; ^b^ = [38]; ^c^ = FVFM Model [19])

The model simulates glaucoma progression in 10,000 hypothetical COAG patients stratified by age (50 and 70 years) and severity of glaucoma at diagnosis. The probability of transition to the next state in the model followed published methodology of Hernández et al. [32] and Briggs et al. [20]; these are driven by the treatment pathways that are used to ameliorate the rate of progression. Again, these are detailed elsewhere [5, 19] but what follows is a short description of the principles underpinning them.

People newly diagnosed with COAG are offered ‘pharmacological treatment’ and this is denoted treatment pathway 1. Patients with COAG who are at risk of progressing to visual impairment despite this first line treatment are offered intensified treatment which might be surgery with pharmacological augmentation. Typically, this would only be done after an observing evidence of disease progression. It is this information that might be yielded earlier by *proposed practice*. In the FVFM model this intensified treatment pathway is denoted as 2 or 3. For our purposes the former would typically be combinations of alternative pharmacological treatments or ‘laser treatment’ whereas the latter would be trabeculectomy with pharmacological augmentation. To model the decision making process behind treatment allocation and its impact upon the probability of transition to worse states of disease, two ophthalmologists with a specialist interest in glaucoma, were consulted to construct simplified treatment pathways that patients would face in a NHS hospital setting [19].

For the FVFM model the treatment pathways are used in a time period denoted as *’imperfect information*‘, where the managing clinician is ‘unaware’ of the patient’s true rate of VF progression, simply because they have not been monitored closely enough. After a defined number of VF tests, we identify the patient’s progression rate, and then enter into a time period defined as ‘*perfect information’*. The clinician now has the opportunity to continue to provide the patient with the existing degree of treatment, or to intensify it. These pathways are linked by a series of decision nodes detailed in the FVFM model. As an example, a *younger patient* entering into glaucoma care at health state 1 (mild damage) and defined as being at low risk of progression would receive treatment pathway 1. If the patient was subsequently defined as having a fast rate of progression then they would be moved to 3rd line treatment but only when the clinician has ‘perfect information’. This functionality was built into the model in order to reflect the resource reallocation that occurs once the clinician identifies those patients who are potentially undertreated. This temporal improvement in patient management is what underpins this study, as the more expedient allocation of efficient treatment modalities differentiates the *proposed practice* from *current practice*. However, this reallocation comes at a cost and this is described briefly below.

A key component of the cost-effectiveness of *proposed practice* is the cost of additional resources for more VF testing. After all, this is seen as the main barrier for implementing increased surveillance and more examinations [23]. Costs were sourced from the reference costs [39] and along with the costs of treatment, (derived from a study reported by Traverso et al. [10]) are taken directly from those used in the FVFM model. (Costs for extra VF testing associated with *proposed practice* did not consider personal costs to the patient such as travel or absence from work for extra clinic appointments.)

Of course a key driver of the cost-effectiveness of the *proposed practice* is the quality-of-life improvement gained from reducing the chances of VF loss and visual impairment. In this study, utility weights associated with each health state were derived from those developed and implemented by Burr and colleagues [31, 40]. Consequently, those defined with mild, moderate, severe disease and visual impairment were attributed a utility of 0.8015, 0.7471, 0.7133 and 0.5350 per year respectively.

### Model analysis

**Fig. 3:**
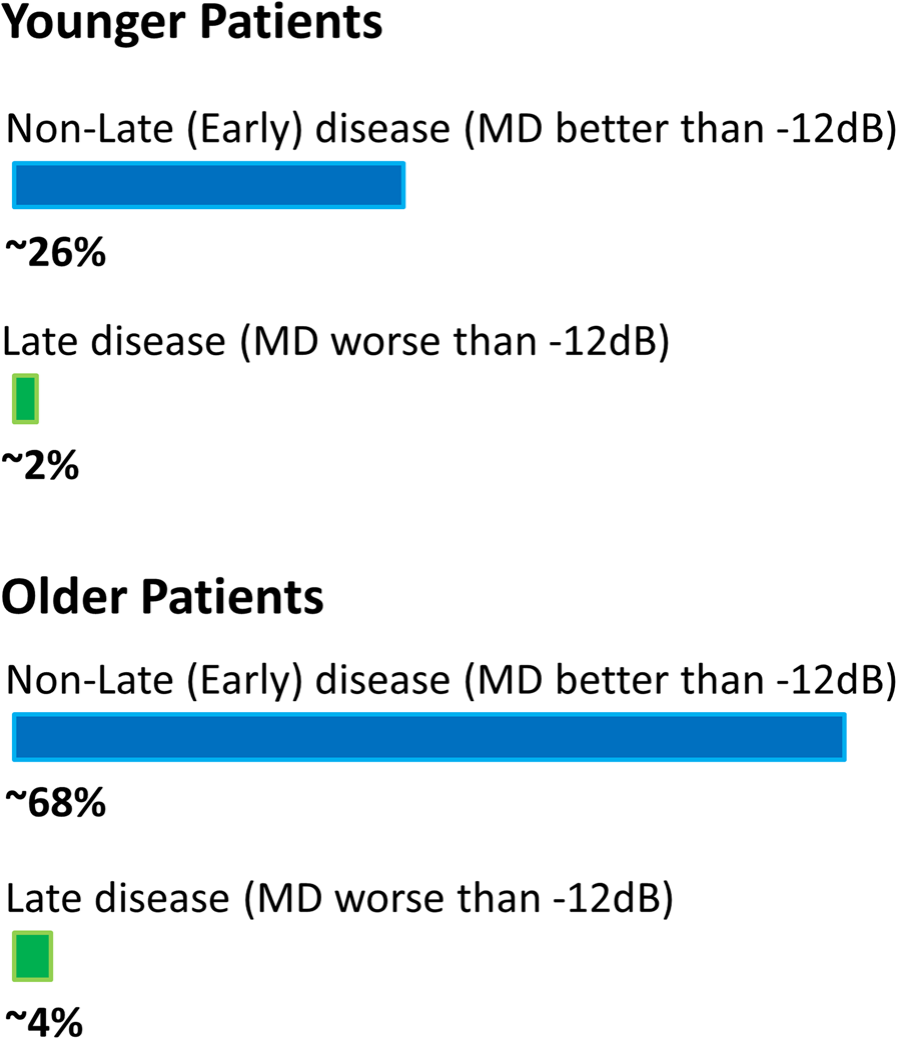
An illustration of the subgroup stratifications used for further cost-effectiveness analysis. Patients were stratified by merging health state groups into what we loosely describe as ‘Late’ disease (severe or worse VF loss in the better eye) or ‘Non-Late’ (‘Early’) Disease (mild and moderate VF loss in better eye)). The former would be patients diagnosed with a level of vision loss that would likely be incompatible with the VF component for legal fitness to drive in the UK [34]. Age distribution was taken directly from that used in the FVFM model. In the model proposed practice was provided to each of the four individual groups in turn with the remaining groups being allocated to the current practice

Our main outcome measure was the ICER derived by *proposed practice* as an alternative to *current practice* as applied to all newly diagnosed patients (full model). A further outcome measure was the years of healthy vision saved with *proposed practice* compared to *current practice*. We then stratified the patient into four groups as described in Fig. 3. Each of the four groups was modelled separately to receive *proposed practice* while all other patients would receive *current practice*. The model results, with the ICER being the primary outcome, were then used to test the hypothesis that applying *proposed practice* to a specific group of patients would be more cost-effective than making it available to all newly diagnosed patients.

In the FVFM model we did not include indirect costs of severe visual impairment from COAG. These are governmental and societal costs for supporting a visually impaired person, such as visual rehabilitation, social services, or local authority care rather than costs of blindness to the individual. Estimating these costs is problematic, country dependent and tricky to establish [41]. Still, some useful estimates are available [8]; these costs were inflated to 2015 levels using the retail price index and were identified as ranging between £1,375 and £17,100 for the first year of blindness and £1,325 and £16,800 for each subsequent year thereafter. We incorporated the most conservative estimate from the range identified because of the uncertainty of the estimates as applied to glaucoma blindness. As such, a modest cost of £1,777 was used in the updated model to represent the economic burden of progression to visual impairment.

Sensitivity analyses were performed in order to examine how parameter uncertainty interacted with model outcomes [42, 43]. Preliminary sensitivity analysis was performed at the earliest stages of model development in order to facilitate the understanding of how inputs interact with model outcomes. One-way deterministic sensitivity analysis (DSA) and probabilistic sensitivity analysis (PSA) were performed on the outputs generated by the Markov model once rationality in these outputs was assumed. From the derived ICERs, incremental cost-effectiveness planes were constructed and from these cost-effectiveness acceptability curves (CEACs) were drawn indicating the probability of *proposed practice* being accepted at given levels of willingness to pay [20, 44].

Ethics approval for this modelling exercise was not required. Access to the non-identifiable patient data summarized in Table 1 was granted by the Caldicott Guardian at each participating centre and is described elsewhere [25, 38]. Subsequent analyses of the data, including that done in this work, were approved by a research ethics committee of City University London.

## Results

In total, 10,000 patients were simulated to enter into the health economic model with a positive cost differential of £298 per patient identified between *proposed practice* and *current practice* (Table 2). This implies higher costs with *proposed practice* but this corresponds with a positive utility differential (0.014 QALYS per patient). Consequently an ICER of £21,392 per QALY was derived for *proposed practice*, a figure within the hypothetical NICE ceiling ratio of £30,000. Furthermore, a total of 785 visual impairment years were saved as a result of increased early monitoring associated with the *proposed practice* across the 25-year time horizon. These results are relevant to applying *proposed practice* to all newly diagnosed patients. Table 2 summarises the results for the scenarios when *proposed practice* is allocated to four specific subgroups of patients.

**Table 2:** ICERs produced once the proposed practice was provided to specific subgroups stratified by age and glaucoma severity

Age subgroup	Severity subgroup	Incremental costs	Incremental utility	ICER
All	All	£298	0.014	£21,392
Younger patient	Early	£306	0.021	£14,797
	Late	£3,251	0.049	£66,219
Older patient	Early	£287	0.014	£21,024
	Late	£4,170	0.030	£138,891

The best ICER associated with *proposed practice* was yielded from the younger cohort diagnosed with early (to moderate) stage VF loss in their better eye. Worse ICERs, incompatible with hypothetical willingness to pay thresholds, are returned for those patients that are already at an advanced disease state on diagnosis in their better eye.

After annual costs of visual impairment (£1,777 per year) were incorporated into the model, an incremental cost of £159 per patient (incremental utility of 0.14) was identified between *proposed practice* and *current practice.* There is no change in terms of incremental QALYs given that societal costs of visual impairment do not impact upon the patient themselves, so this yielded an ICER of £11,382 per QALY being derived for *proposed practice*. This represents a significant reduction in the ICER compared to result without visual impairment costs added. The latter were then varied to identify the threshold for cost neutrality between the *current practice* and *proposed practice* across both the full simulation. Under the full simulation, a value of £3,798 was identified as the required costs of visual impairment to equate *proposed practice* to *current practice*.

### Sensitivity analysis

**Fig. 4:**
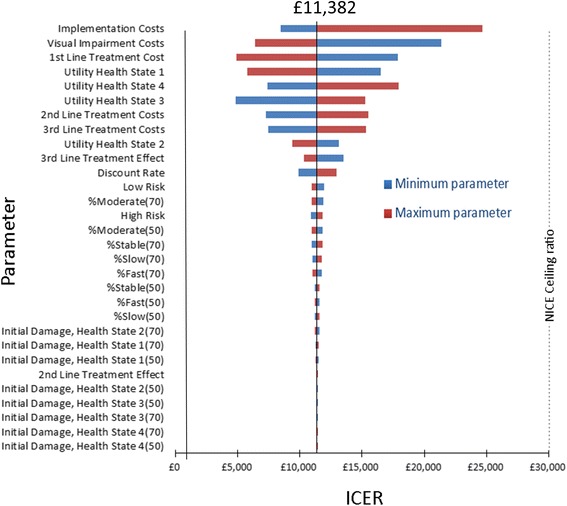
Tornado Diagram measuring the impact in variation in parameters for the health economic model with included visual impairment costs (ICER = £11,382). Maximum and minimum limits for parameters were identified. ICERs were derived and ordered in terms of impact (greatest to lowest ICER variation)

DSA results are presented in a Tornado diagram for the full simulation (Fig. 4). The horizontal axis is the outcome (the ICER for allocating *current practice* to all newly diagnosed patients); along the vertical axis, parameters are ordered and horizontal bars represent the outcome range associated with the specified parameter’s range (maximum and minimum value limits impact upon ICERs). For all parameters, outcomes were sorted in order of ICER impact. The uncertainty surrounding the implementation cost parameter and the visual impairment cost parameter resulted in the highest ICER variations but neither were sufficient enough to push the ICER beyond the £30,000/QALY ceiling ratio. For example, the maximum identified limit for implementation costs (£2.26 m) resulted in an ICER of £24,600. Tellingly, the next most important parameters were the treatment costs and utility health states followed by the distributions of the existing damage. Progression rates were found to have very little impact despite being varied by 10 % in either direction.

**Fig. 5:**
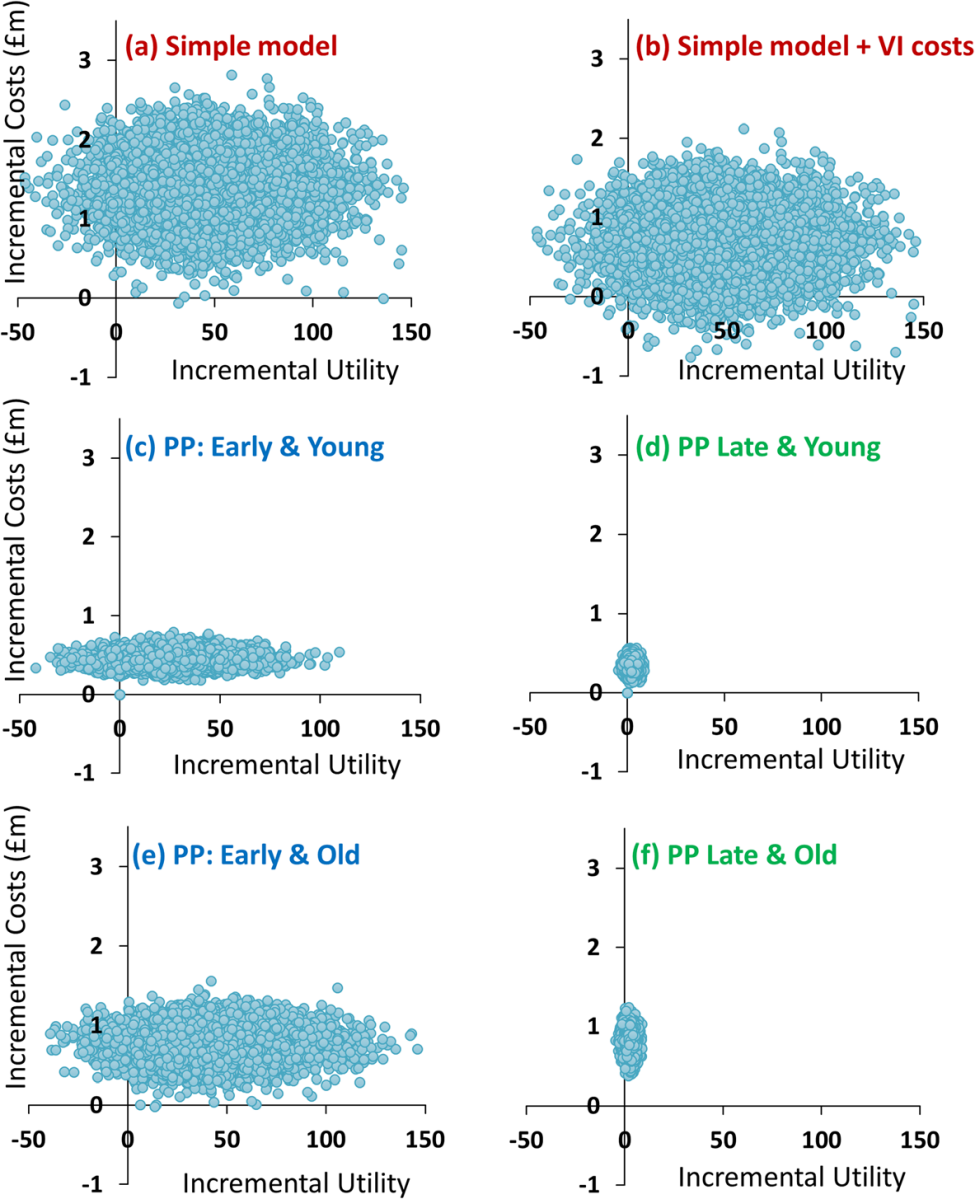
Cost-Effectiveness Planes for the different subgroups analysed

Unsurprisingly, in the PSA (see Fig. 5), greater cost-effectiveness was observed when costs of visual impairment were included (b) compared to when it was not (a). The observations in (b) are lower on the plane (indicating lower costs) with little change in the width of the observations (indicating similar effectiveness). *Proposed practice* in younger patients with early glaucoma (c), placed observations significantly lower on the plane than in the simple model (a), indicating a significant improvement on cost-effectiveness. Patients (both young and old) with advanced glaucoma yielded a compressed cluster of observations. Simply, the model is inferring that those with late glaucoma have less vision to save; therefore, less incremental utility can be derived. For older patients with early glaucoma (e), observations were more spread across the cost-effectiveness plane suggesting greater likelihood of utility gain given their greater preserved vision.

**Fig. 6:**
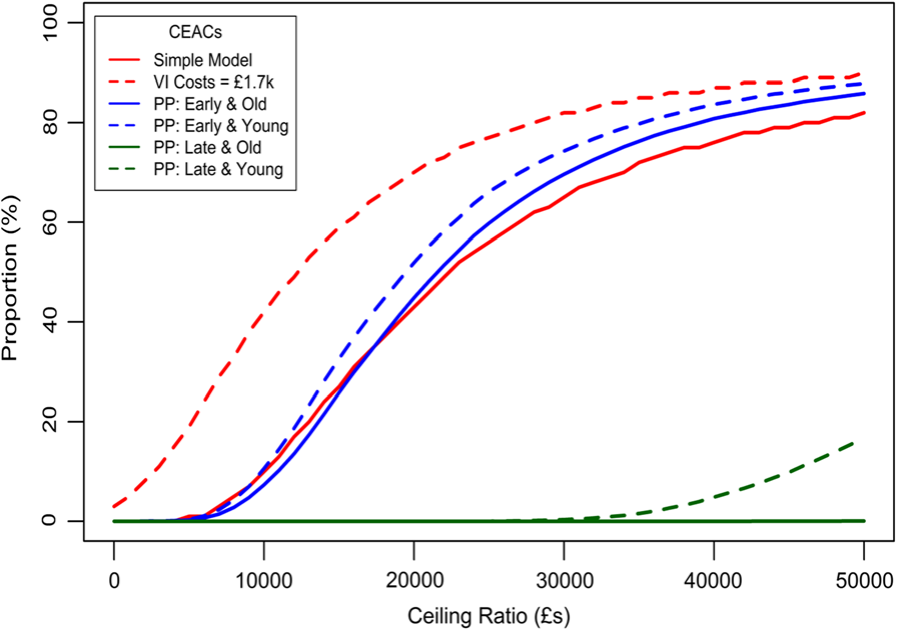
Cost Effectiveness Acceptability Curves across the subgroups analysed

CEACS were derived from these simulations (see Fig. 6). Willingness to pay for each QALY gained was varied from £0 to £50,000 and the proportion of simulations deemed acceptable at this level were recorded. Similar shaped CEACs were observed for the model with and without the visual impairment costs added. However, the shift of the CEAC to the left for the model with visual impairment costs included indicates an increased probability of acceptance of this scenario. At the £30,000 per QALY ceiling ratio, the proposed practice was acceptable 82 % of the time when these indirect costs were modelled whilst only 65 % of the time when they were not. When *proposed practice* was provided to patients with early glaucoma, there was less deviation from the simple model with 70 % (old) and 74 % (young) being observed to be acceptable at the £30,000 per QALY ratio. CEACs trail close to zero for the patients diagnosed with late disease indicating a significant lack of cost-effectiveness likelihood for these subgroups.

## Discussion

This modelling exercise primarily sought to examine whether increased VF monitoring at the earliest stages of disease identification in COAG (i.e. six VFs in the first 2 years after diagnosis) would be cost-effective compared with the assumed *current practice* of one VF per year. An ICER of £21,392 indicates that the *proposed practice* is a cost-effective strategy for all patients given a hypothetical £30,000/QALY NICE acceptability ceiling ratio. So these health economic findings support the EGS guideline recommendation of undertaking 6 VFs in the first 2 years after glaucoma diagnosis.

Introduction of costs of visual impairment further increased the cost-effectiveness of the *proposed practice*. A cost of visual impairment threshold of £3,798 per year was identified as the minimum value required ensuring that the *proposed practice* would equate to *current practice* in terms of costs across the full 25 year time horizon of the model. Put another way, if cost of visual impairment per year is assumed to be greater than £3,798 per year then, *proposed practice* is preferable to *current practice*. This figure sits at the lower end of the distribution of the costs estimated in the Meads et al. study (£1,325 to £16,800 per year throughout the duration of patients residual lifetime) [8]. Moreover other studies have estimated costs of visual impairment to be significantly greater. For example Lafuma and colleagues reported a value of €13,674/year in the UK in 2006 (equating to about £11,000/year when inflated to 2015 values and converted to pound sterling) and Burr et al. suggested the figure could be as high as £40,000/year [33, 45]. Therefore, if we were to use the values found in these alternative studies, the *proposed practice* would be the cheaper long term patient pathway compared to *current practice* due to the costs saved by reducing the amount of patients progressing to visual impairment over the 25 years (an ICER of £11,383 per QALY). We think this is a significant finding. Moreover, our report should stimulate more research into the hidden costs of burden of sight loss and encourage other researchers to include them in their health economic models when studying conditions that lead to sight loss. We haven’t included them all. For instance, ‘hidden’ costs arise because people with visual impairment tend to have longer hospital stays for co-existing morbidities [46, 47] whilst risk of falling is higher for the visually impaired, inevitably leading to more ‘hidden’ costs [48].

Within this modelling exercise we loosely defined patients to be in a severe disease state if the MD is worse than -12 dB in the better eye. This threshold is not entirely arbitrary because it approximately equates to a patient failing the VF component of legal fitness to drive in the UK [34] and has been used in staging disease severity in COAG before. In those patients with VF loss better than this threshold, proposed practice seems particularly cost-effective. Patients with sight loss worse than this threshold would likely be on maximum therapies anyway and our model suggests it would be less cost-effective to monitor them closely at the outset. This might appear controversial but it simply reflects the limited treatment options in late stage glaucoma. Interestingly the idea that surgery ought to always be the primary treatment option for people diagnosed with advanced glaucoma is being tested in an on-going large randomised trial in the UK (https://www.tagsstudy.co.uk/).

*Proposed practice* is more cost-effective in younger patients (Table 2). This is unsurprising because the costs of *proposed practice* are more likely to be recovered for a person with longer residual life expectancy, with the economic argument of early investment in preserving future vision. More intensive monitoring of these patients is obviously worthwhile in order to establish speed of loss and improve their clinical management. Yet recent research indicates that frequency of monitoring in clinics in England does not vary by the age of the patient (or rate of loss or disease severity for that matter) - younger and older patients simply get the same diet of VF testing [25]. Therefore, and at the very least, our model provides evidence for the potential cost-effectiveness of stratifying patients to more or less monitoring and this is an important conclusion from our work. A prospective research study examining this issue is recommended. At the moment, there is a tendency to have a ‘one size fits all’ approach to monitoring the diagnosed patients and this is likely a suboptimal method for monitoring large cohorts of patients. In addition, previous modelling work has indicated clustering tests at the beginning and end of the initial 2-year period after diagnosis, rather than evenly spaced VFs, will identify more rapid VF progression with fewer false positives. [18] Again, a prospective study examining the health economics and benefits to patients of using this approach is recommended.

Sensitivity analysis identified implementation costs as the most important parameter impacting upon the ICER, resulting in ICERs ranging from £8,400/QALY at its minimum value to £24,700/QALY at its maximum value. A full costing study examining the range of values is clearly required to truly ascertain whether this maximum value is accurate or if there is already sufficient excess capacity to allow for the *proposed practice* (the minimum modelled assumption). The second most important parameter within the sensitivity analysis was the costs of visual impairment. The minimum assumption of costs to society equalling £0 resulted in a maximum ICER of £21,400. However this perceived minimum limit is unlikely to be representative in the real world especially given what an economist would refer to as the negative externalities associated with glaucoma. We therefore reiterate the message about the need for further studies to estimate these costs more precisely [33]; without them the predictions from health economic models of age-related eye disease will always lack precision. Costs associated with the 1^st^ line of treatment modelled within this study were identified as the third most important factor within the Tornado analysis with the lowest assumed value (£389) resulting in an ICER of £17,800. As *proposed practice* accelerates the time it takes to get an ‘upgrade’ in treatment modality provision, patients are therefore moved away from the 1^st^ line of treatment at an increased rate. If costs for the 1^st^ line of treatment are relatively low, it becomes less economically efficient to move to the 2^nd^ and 3^rd^ line of treatment, therefore making the *proposed practice* less cost-effective. This result points to the need for better data on true costs of treatment for glaucoma and this is worthy of further research, especially the ‘one off’ cost for a surgical intervention compared to long-term use of medical therapy.

A comprehensive study comparing resource utilisation in the management of COAG in two cities in Finland yielded interesting results that contradict some of the findings in our work [49]. In particular more intensive patient monitoring, over an 11-year period, did not seem to benefit patients. Results, albeit based on a retrospective analysis of data from relatively small populations, indicated increased resource allocation did not lead to measurable benefits to patients in terms of less glaucoma-induced visual disability or self-reported quality of life. It is noteworthy that increased resource allocation was due mainly to increased treatment costs rather than increased monitoring *per se*. This report, like ours, concluded a prospective study is required to truly examine the benefits of more or less monitoring in groups of patients with COAG.

Intensive monitoring of a chronic disease and acting on detection of progression becomes more important if there are a range of options to intensify treatment; this isn’t really the case with COAG. For example, in our model we only had three lines of ranked treatment options; a realistic scenario given lack of treatment options in COAG. After all, the only modifiable risk factor for disease progression is reduction of IOP. Some carefully done health economic modelling work has demonstrated increased cost effectiveness by aiming for a low IOP (standard IOP < 15 mmHg) in all COAG patients after diagnosis; this in turn would remove demand for intensive monitoring and reduce the need for frequent VF testing. [50–52]

### Limitations of the study

It is difficult to accurately model real world clinical decision making. Here clinical decision pathways were developed in consultation with two practicing glaucoma specialists [19]. Decision making varies from clinician to clinician however and it is possible that a clinical review panel made up of different ophthalmologists could have resulted in alternative decision nodes being constructed. In addition, we only implemented three possible treatment lines for simplicity but in reality there are significantly more possible variations in treatment lines that the patients could undergo. Moreover, our model did not account for impact of co-pathology on outcomes for glaucoma therapy; this thorny issue awaits a further study.

Critically our model does not consider the effect of false positive decisions on VF progression. After all, it has been shown that increasing VF testing will inevitably affect specificity [18]. Therefore, with *proposed practice* patients may receive intensified treatment when it is not required and our model is not adjusted for this cost. Our model has also made many necessary assumptions about the costs of additional visual field testing. Moreover it assumes that all patients can provide reliable VF results when many do not.

Our model only considered VF monitoring and not the other assessments that need to be made during glaucoma follow-up. For example, there are certainly interesting open research questions about frequency of monitoring and imaging the optic nerve or retinal nerve fibre layer as surrogates for disease progression in COAG, either in tandem with VF assessment or alone. Our model also assumed the VF changes in a linear fashion only. This is reasonable given work done in this area [53] but deterioration to noticeable binocular vision loss may be more suddenly noticed in patients [2].

One of the main limitations of this study consists of implementing health state utility values derived from a small number of people: 37 patients with mild COAG (0.8015), 14 patients with moderate COAG (0.7471) and just 9 patients with severe COAG (0.7133). Other health economic models of health service delivery of COAG have used different estimates for comparable health state utility values [49, 51, 54]. There does not seem to be a consistent approach, or optimal study design, for generating these values. We suggest this represents a significant gap in knowledge for an important component of health economic modelling for COAG.

An economic evaluation using discrete event simulation might also model the process more accurately and this has been used elsewhere [52]. Still, such models are complex and difficult to interpret and a Markov model structure offers simplicity and transparency.

Our model is likely also limited by the way in which disease severity was categorised - more work is needed to establish meaningful stratification of functional loss in glaucoma. Our results only considered a summary measure from the VF. Research has shown that an index like MD does not capture location and spatial extent of VF loss in patients [2]. For example, two patients with the same MD might have different visual function. Moreover, there is debate about using a measure of binocular VF loss and aligning this with utilities [35, 36]. Finally, our model does not capture the co-morbidities of patients; this could be concomitant eye disease or other chronic conditions.

### Future research

Measuring impact of visual function loss on quality of life requires further study in order to test the clinical- and cost-effectiveness of health service delivery of COAG [55]. Further research to quantify the costs of sight impairment is also a priority. Also, little is known about how patients adapt to gradual sight loss in glaucoma and this subject is worthy of further study; we speculate that this could have a significant bearing on estimating utilities in health economic models for COAG [56]. Indeed we suggest there are clear uncertainties surrounding the utilities in these models despite exemplar studies attempting to derive meaningful values [40, 54]. New research should look at the precision and accuracy of these values. Furthermore, whilst a range of ‘theoretical’ implementation costs were examined in the sensitivity analysis of our model results, it was beyond scope to examine in detail the costs associated with implementing *proposed practice;* this clearly ought to be the subject of further research along with consideration of the thoughts on increased testing of patients and clinicians [23, 57]. Consideration of innovative and affordable health service delivery redesign is likely to be a wider debate that needs to be addressed too, as has been recently suggested for people with ocular hypertension [58, 59].

## Conclusion

Results from this modelling exercise indicate the health economic benefits of intensifying monitoring of patients after they have been newly diagnosed with COAG. Increasing the number of VF examinations to better determine those patients’ that are rapidly losing vision appears to be cost-effective; this might be particularly true for younger patients. A study on the resource implications for glaucoma follow-up and costs of sight impairment from COAG would be worthwhile. A prospective study of different follow-up patterns, especially stratified among different patient groups is recommended.

## Abbreviations

CEAC: Cost-effectiveness acceptability curves
COAG: Chronic open angle glaucoma
DSA: Deterministic sensitivity analysis
EGS: European Glaucoma Society
FVFM: Frequency of visual field monitoring model
ICER: Incremental cost-effectiveness ratio
IOP: Intraocular pressure
MD: Mean deviation
NHS: National Health Service
NICE: National Institute for Health and Care Excellence
PSA: Probabilistic sensitivity analysis
QALY: Quality-adjusted life year
UK: United Kingdom
VF: Visual field
